# *SOX1* Promoter Hypermethylation as a Potential Biomarker for High-Grade Squamous Intraepithelial Neoplasia Lesion and Cervical Carcinoma: A Meta-Analysis With Trial Sequential Analysis

**DOI:** 10.3389/fgene.2020.00633

**Published:** 2020-07-31

**Authors:** Jin Huang, Hong Gao, Hong-Zhuan Tan

**Affiliations:** ^1^Department of Epidemiology and Health Statistics, School of Public Health, Central South University, Changsha, China; ^2^School of Nursing, University of South China, Hengyang, China

**Keywords:** cervical cancer, DNA methylation, *SOX1*, diagnosis, meta-analysis

## Abstract

**Background:** DNA methylation has been widely assessed as a potential biomarker for the early detection of cervical cancer (CC). Herein, we assessed the associations of SOX1 promoter hypermethylation with squamous intraepithelial lesion and CC.

**Methods:** Published studies and genome-wide methylation datasets were searched from electronic databases (up to April 2019). The associations of SOX1 hypermethylation with high-grade squamous intraepithelial lesion (HSIL) and CC risks were evaluated by odds ratios (ORs) and 95% confidence intervals (CIs). The summary receiver operator characteristic test was used to assess the diagnostic value of the SOX1 promoter hypermethylation of CC and intraepithelial neoplasia type III or worse (CIN3+). Trial sequential analysis (TSA) was performed to evaluate the stability of results and estimate the required information size (RIS).

**Results:** In this meta-analysis of 17 published studies, the SOX1 methylation rates increased among low-grade SIL (LSIL, 27.27%), HSIL (40.75%), and CC (84.56%) specimens. Compared with control specimens, SOX1 promoter hypermethylation progressively increased the risk of HSIL by 4.20-fold (*p* < 0.001) and CC by 41.26-fold (*p* < 0.001). The pooled sensitivity of SOX1 methylation was estimated to be 0.85 (95% CI: 0.81–0.88) in differentiating patients with CC, corresponding to a specificity of 0.72 (95% CI: 0.69–0.75) and an area under the curve (AUC) of 0.93. Furthermore, the pooled sensitivity of SOX1 methylation was estimated to be 0.75 (95% CI: 0.72–0.78) in differentiating patients with CIN3+, corresponding to a specificity of 0.71 (95% CI: 0.69–0.73) and an AUC of 0.84. The pooled results of TCGA and GEO datasets showed that all CpG sites in SOX1 were associated with CC and 16 of 19 CpG sites were associated with HSIL. The results of TSA illustrated that the size was sufficient and significant associations were observed.

**Conclusion:** This meta-analysis indicated that SOX1 promoter hypermethylation might have a potential value in the clinical diagnosis of CC and CIN3+.

## Introduction

Cervical cancer (CC), a common gynecological malignant tumor, is a major disease that seriously threatens women's health (Wright et al., 2019). According to 2018 global cancer statistics, CC is the fourth leading cause of cancer death with 569,847 new cases and 311,365 deaths per year (Bray et al., 2018). More than 80% of these cases occur in developing countries, in which CC is the leading cause of cancer deaths (Di et al., 2015). CC can be classified into squamous cell carcinoma (SCC) and adenocarcinoma (AdC) (Mpunga et al., 2019). The development of CC is characterized as a progressive process from normal epithelium to squamous intraepithelial lesion (SIL) and eventually to invasive carcinoma. SIL, a precursor lesion of CC, consists of low-grade SIL (LSIL) and high-grade SIL (HSIL) (Terra et al., 2007; Chen et al., 2018). Cervical intraepithelial neoplasia (CIN) is a premalignant lesion of cervical cancer, histologically divided as CIN1, CIN2, and CIN3 (Hurtado-Roca et al., 2020). According to the categorization of the 2001 Bethesda System, the diagnosis of LSIL including productive HPV infection, CIN1 and mild dysplasia, while the category of HSIL including CIN 2 or 3, moderate and extensive dysplasia and carcinoma *in situ* (CIS) (Solomon et al., 2002).

DNA promoter methylation, as a common epigenetic modification in humans, is closely related to the genomic stability and transcriptional silencing of tumor suppressor genes (TSGs) (Herman and Baylin, 2003; Robertson, 2005). Gene silencing by promoter hypermethylation is related to the development and progression of human cancers, including CC (Virmani et al., 2001; Kekeyeva et al., 2006). *SOX1* is an important TSG that regulates the expression of genes involved in cell proliferation (Guan et al., 2014) and invasion (Song et al., 2016). At present, *SOX1* promoter methylation is related to the occurrence and development of various tumors. Lai et al. (2008) reported for the first time that *SOX1* and five other genes are more frequently methylated in SCC tissues than in their normal controls. Thus, *SOX1* methylation could be a good biomarker to distinguish HSIL from non-specific cytological changes and the normal cervix in liquid-based cytology (Apostolidou et al., 2009). Thereafter, an increasing number of studies focused on the relationship of *SOX1* promoter methylation with the screening of CC or SIL. It has been generally considered that cervical cancer and precursor lesions were usually caused by high-risk type of human papillomavirus (HPV). And, methylation level of *SOX1* was significantly higher in the presence of viral infection of high-risk HPV. The prevalence of CIN 2+ was very low in cases without high-risk HPV infection and *SOX1* unmethylated events. The prevalence of CIN 2+ were slightly higher when one of the factors was present (HPV infection or *SOX1* methylation) but significantly higher when both factors were simultaneously considered (Rogeri et al., 2018). Another meta-analysis assessed the relationship of CC development with the promoter methylation of *SOX1* together with *PAX1* (Chen et al., 2016), but only Asians were involved in this research. Studies on the methylation level of *SOX1* in different carcinogenesis stages from precancerous lesions to CC are still lacking.

Therefore, we performed a meta-analysis to assess the correlation between *SOX1* promoter hypermethylation and CC or SILs. We assessed the association of *SOX1* promoter hypermethylation and SILs and CC by combining the data of 17 published studies and then by combining six genome-wide quantitative methylation datasets from online databases, including TCGA and GEO.

## Materials and Methods

### Search Strategy for Published Studies

All relevant studies were initially searched from the PubMed, Web of Science, EMBASE, China National Knowledge Infrastructure, and Wanfang databases, and the search time was up to April 2019. The search was performed by using following keywords: “cervical cancer” or “cervical carcinoma” or “cervical tumor” and “*SOX1*” and “methylation” or “hypermethylation” or “epigene^*^.” Reference lists in retrieved articles and relevant reviews were also manually retrieved. This meta-analysis was reported in accordance with the Preferred Reporting Items for Systematic Reviews and Meta-Analysis (PRISMA) 2009 guidelines (Supplementary Table 1).

### Eligibility Criteria for Published Studies

Studies meeting the following criteria were included in this meta-analysis:

1. the association of *SOX1* methylation with CC or squamous intraepithelial lesion was evaluated;
2. sufficient data were provided to calculate odds ratios (ORs) and 95% confidence intervals (CIs);
3. matched controls were included;
4. written language used was English or Chinese.

Studies meeting the following criteria were excluded:

1. reviews, meta-analysis, meeting abstracts, letters, or case reports;
2. control group and sample sizes were unclear;
3. *in vitro* or *ex vivo* experiments of cell lines or animals.

### Data Extraction of Published Studies

All included studies were independently extracted by two authors. The following information of each eligible study was extracted: first author's name, publication year, ethnicity, country, methylation detection methods, materials, source of controls, sample size-involved diseases (LSIL, HSIL, CC, CIN3+/CIN2–), clinicopathological features (age at diagnosis, HPV infection, histological type, federation International of Gynecology and Obstetr (FIGO) stage, and lymph node metastasis), and quality of studies.

### Data Extraction of GEO and TCGA Datasets

Genome-wide methylation profiles of the TCGA CESC project were obtained. Quantitative methylation datasets were initially searched from the GEO database (https://www.ncbi.nlm.nih.gov/gds/) by using the following keywords: “cervical cancer” and “methylation” and “*Homo sapiens*.” Methylation signals of the datasets above were detected by Illumina HumanMethylation 27 or 450 k Beadchip. The level of methylation at each CpG island site is expressed as a beta value, which is the ratio of quantile-normalized methylation intensity to total locus intensity (methylated and unmethylated).

### Trial Sequential Analysis

Trial sequential analysis (TSA) was performed on the *SOX1* methylation frequency of the control group and patients with CC, HSIL or CIN3+ to evaluate the stability of results and the required information size (RIS). TSA 0.9 software (Copenhagen Trial Unit, Denmark, http://www.ctu.dk/tsa/) was applied to assess statistical significance. In this meta-analysis, the type I error was set as 5%, and the type II error rate was set as 20% with a statistical test power of 80%. The cumulative Z-curve crossed the trial sequential monitoring boundary or the required information size, suggesting that the statistical evidence is firm for this meta-analysis. Otherwise, additional studies are essential to reach a conclusive result. If the Z-curve did not cross any boundary, then no significant association existed. If the Z-curve crossed the traditional boundary and the trial sequential monitoring boundary, then the sample size was large enough and a significant association existed.

### Quality Assessment

The quality of included articles and datasets was independently evaluated by two authors (JH and JYL) according to a predefined system derived from the REMARK (Altman et al., 2012) and BRISQ (Moore et al., 2011) guidelines. Eighteen items were considered as quality components, including study design, study population, biospecimen information, methylation detection, clinicopathological features, and result analysis (Supplementary Table 2). Articles or datasets covered more than 11 items were considered high quality.

### Statistical Methods

The *SOX1* promoter methylation rates in the LSIL, HSIL, CC, and control specimens were calculated by the inverse variance method. Chi-square test for trend was used to compare the methylation frequency in the control group, LSIL, HSIL, and CC specimens. Pooled odds ratios (ORs) and their 95% CIs were calculated to assess the association of *SOX1* promoter hypermethylation with LSIL, HSIL, CC, CIN3+, and HPV. Heterogeneity of eligible studies was determined by the Cochran's *Q*-test and *I*^2^ statistic. The random-effect model was applied to pool the results when significant heterogeneity (*I*^2^ value larger than 50% or *P*_Q−test_ smaller than 0.1) was present; otherwise, the fixed-effect model was applied. The bivariate meta-analysis model was performed to estimate the sensitivity, specificity, diagnostic odds ratio (DOR), positive likelihood ratio (PLR), and negative likelihood ratio (NLR) and to generate the summary receiver operator characteristic (SROC) curves. Subgroup analyses and meta-regression were conducted to further explore the possible source of heterogeneity based on ethnicity, source of controls, materials, published year (≥2015 and <2015), and quality of studies. Funnel plots and Egger's test were conducted to evaluate the potential publication bias, and *P*_Egger_ ≤ 0.05 implied the existence of publication bias (Egger et al., 1997). Statistical analysis was carried out using RevMan 5.3 (The Nordic Cochrane Centre, The Cochrane Collaboration), Stata 15.0 (Stata Corporation, College Station, TX, USA), and Meta-disc 1.4 (XI Cochrane Colloquium, Barcelona, Spain) software.

## Results

### Characteristics of Included Studies

**Figure 1:**
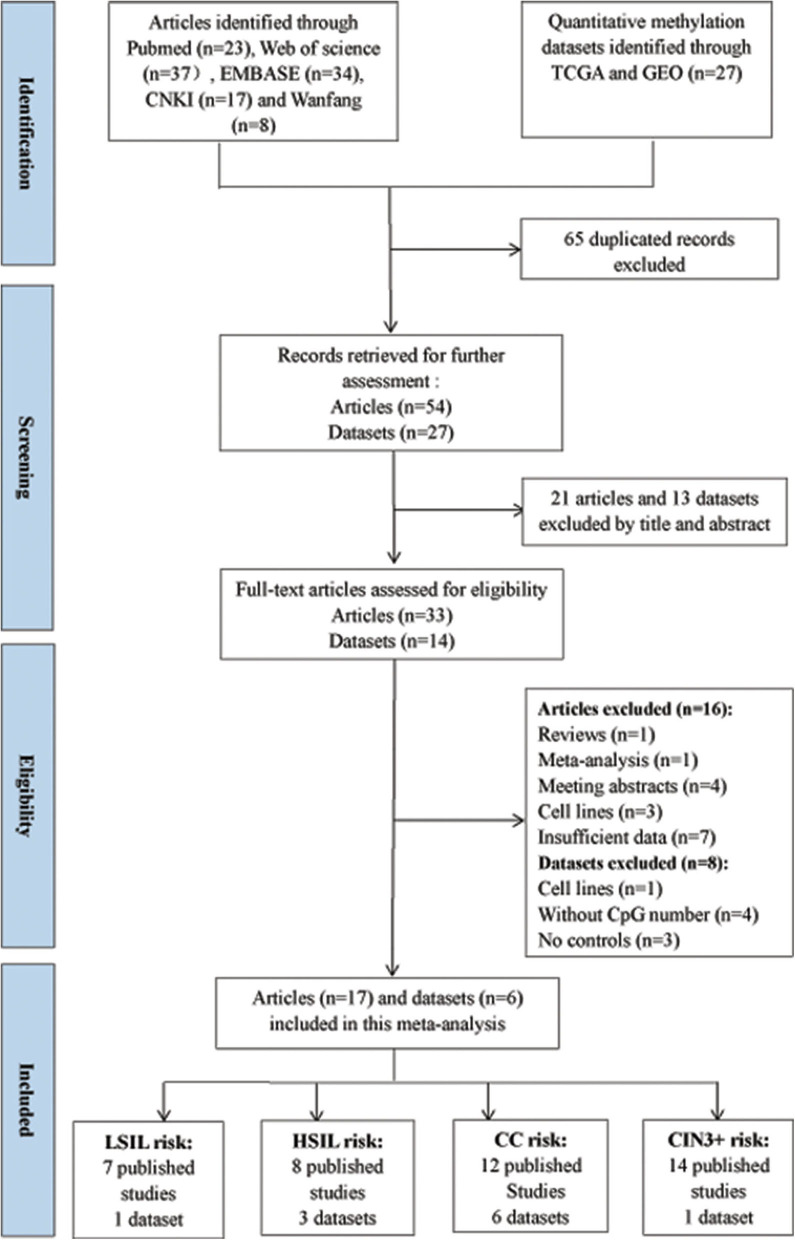
Flow diagram for the procedures of eligible studies selection in this meta-analysis.

According to the definitions of the 2001 Bethesda System, the category of LSIL encompassed cytopathic effects of HPV, cervical intraepithelial neoplasia (CIN) 1, and mild dysplasia. The category of HSIL contained moderate or extensive dysplasia and CIN 2 or 3. The flow diagram for the procedures of eligible studies selection in this meta-analysis is shown in Figure 1. CC contained SCC and AdC. A total of 54 studies were initially selected without duplicate from the Pubmed, Web of Science, EMBASE, CNKI, and Wanfang databases. Then, 21 studies were excluded after reviewing their title and abstract. Then, 16 published articles were excluded due to review (*n* = 1), meta-analysis (*n* = 1), meeting abstracts (*n* = 4), cell lines (*n* = 3), and insufficient data (*n* = 7).

Finally, 3491 patients with CC or CIN from 17 articles (Lai et al., 2008, 2010, 2014; Apostolidou et al., 2009; Xu et al., 2010; Chang et al., 2014, 2015a,b; Kan et al., 2014; Lin et al., 2014; Liu et al., 2016; Wang et al., 2016; Wu, 2016; Tian et al., 2017; Yuan, 2017; Rogeri et al., 2018; Robert et al., 2019) were included in this meta-analysis. Among these articles, 12 articles including 434 CC patients and 1,029 controls were used to analyze the correlation between *SOX1* promoter methylation and clinicopathological features. Among these 17 articles, 12 used exfoliated cells of cervical specimen to detect *SOX1* methylation status, 3 (Xu et al., 2010; Pun et al., 2015; Wu, 2016) used cervical tissues, and 2 (Chang et al., 2014; Wang et al., 2016) involved both tissues and cervical exfoliated cells. For most of these 14 studies (14 of 17), the detection of *SOX1* promoter methylation was performed by QMSP. Two studies (Chang et al., 2014; Yuan, 2017) performed pyrosequencing and one study (Lai et al., 2008) performed bisulfite sequencing. Thirteen studies were conducted on Asians, three studies (Apostolidou et al., 2009; Wang et al., 2016; Robert et al., 2019) on Caucasians, and one study (Rogeri et al., 2018) on Brazilian. The characteristics of the included studies are summarized in Table 1.

**Table 1:** Characteristics of published studies and quantitative methylation datasets included in this meta-analysis.

**No**.	**Author**	**Year**	**Country**	**Ethnicity**	**Sample size**						**Methylation detection method**	**Materials**	**Source of controls**	**Involved clincopa-tholo gical features**	**Quality scores**
					**Control**	**CC**	**HSIL**	**LSIL**	**CIN2–**	**CIN3+**					
**PUBLISED STUDIES FROM PUBMED, EMBASE, WEB OF SCIENCE, CNKI AND WANFANG DATABASES**															
1	Robert W	2018	Netherlands	Caucasian	189	3	32	11	216	19	QMSP	exfoliated cells	B	-	14
2	Rogeri CD	2018	Brazil	Brazilian	-	-	-	-	276	126	QMSP	exfoliated cells	B	HPV	13
3	Tian Y	2017	China	Asian	81	17	62	13	121	52	QMSP	exfoliated cells	B	-	13
4	Yuan LQ^a^	2017	China	Asian	-	-	-	-	141	118	Pyrosequencing	exfoliated cells	B	-	11
5	Wang R	2016	Netherlands	Caucasian	27	58	106	38	110	119	QMSP	exfoliated cells	B	type	13
6	Wang R	2016	Netherlands	Caucasian	15	12	-	-	-	-	QMSP	tissue	B	-	12
7	Liu	2016	China	Asian	26	31	57	-	53	61	QMSP	exfoliated cells	H	type, size, FIGO	11
8	Wu^a^	2016	China	Asian	51	68	-	-	-	-	QMSP	tissue	B	HPV, FIGO, age	10
9	Chang	2015	China	Asian	-	-	-	-	55	7	QMSP	tissue	B	-	12
10	Chang	2015	China	Asian	-	-	-	-	140	132	QMSP	exfoliated cells	H	-	12
11	Kan	2014	China	Asian	299	4	39	76	382	36	QMSP	exfoliated cells	H	-	11
12	Lai	2014	China	Asian	199	30	62	55	274	72	QMSP	exfoliated cells	B	-	11
13	Chang	2014	China	Asian	22	23	-	-	-	-	Pyrosequencing	exfoliated cells	H	-	12
14	Chang	2014	China	Asian	45	40	-	-	-	-	Pyrosequencing	tissue	H	-	12
15	Lin^a^	2014	China	Asian	-	-	-	-	215	15	QMSP	exfoliated cells	B	age	10
16	Lai	2010	China	Asian	-	-	-	-	103	73	QMSP	exfoliated cells	H	-	12
17	Xu^a^	2010	China	Asian	30	40	15	15	45	55	QMSP	tissue	B	-	10
18	Apostolidou S	2009	UK	Caucasian	-	-	-	-	62	33	QMSP	exfoliated cells	B	-	11
19	Lai	2008	China	Asian	45	108	54	45	-	-	Bisulfite sequencing	exfoliated cells	H	FIGO stage,LN	13
**QUANTITATIVE METHYLATION DATASETS FROM TCGA AND GEO DATABASES**															
20	TCGA		USA	Mix	3	3	-	-	-	-	Illumina HumanMethylation 450K BeadChip		A	-	14
21	GSE99511	2017-2019	Netherlands	Caucasian	28	4	36	-	-	-	Illumina HumanMethylation 450K BeadChip		B	-	11
22	GSE46306	2013-2019	Sweden	Caucasian	20	6	17	-	-	-	Illumina HumanMethylation 450K BeadChip		H	-	13
23	GSE41384	2012-2015	Colombia	Mix	3	3	10	3	-	-	Illumina HumanMethylation 27K BeadChip		H	-	13
24	GSE36637	2012-2015	Belgium	Caucasian	4	5	-	-	-	-	Illumina HumanMethylation 27K BeadChip		H	-	11
25	GSE30760	2011-2015	United Kingdom	Caucasian	15	48	-	-	77	75	Illumina HumanMethylation 27K BeadChip		M	-	12

a *Studies written in Chinese*.

*B, controls with benign cervical diseases; H, healthy controls; A, autologous controls; M, mixed controls*.

### Pooled Rates of SOX1 Promoter Methylation in Patients With LSIL, HSIL, and CC

A total of 253 LSIL, 427 HSIL, 434 CC, and 1,029 controls specimens were included in this meta-analysis. As shown in Table 2, the pooled rates of *SOX1* hypermethylation showed an increasing trend (*p* < 0.001) from the LSIL specimens (27.27%, 95% CI: 4.43%−20.81%) to the HSIL specimens (40.75%, 95% CI: 24.27%−63.27%) and ultimately to the CC specimens (84.56%, 95% CI: 83.59%−93.08%).

**Table 2:** Pooled hypermethylation rates of *SOX1* in LSIL, HSIL and CC specimens.

**Comparisons**	**Studies**	**Specimens**	**Methylation rates (%)**	**95% CI (%)**
Control	19	1029	12.16%	4.43–20.81
LSIL	7	253	27.27%	11.46–40.35
HSIL	8	427	40.75%	24.27–63.27
CC	12	434	84.56%	83.59–93.08

### Correlation Between SOX1 Promoter Methylation With SIL and CC Risk

**Figure 2:**
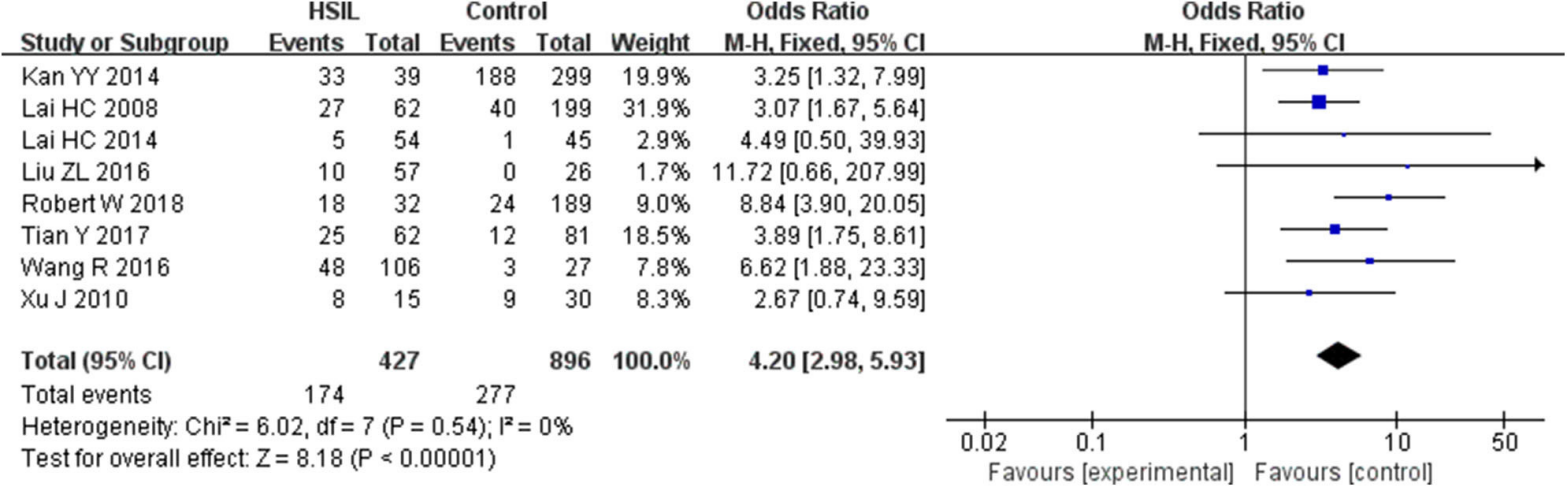
Forest plots for associations of *SOX1* promoter hypermethylation with the risk of HSIL. The squares represent the ORs for individual studies. The size of the square reflects the weight of included studies. Bars represent the 95% confidence intervals (CIs). The center of the diamond represents the summary effect size. Abbreviations: HSIL, high-grade intra-epithelial lesion; ORs, odd ratios.

Seven studies including 253 patients with LSIL and 870 controls were included to assess the effect of *SOX1* promoter methylation on LSIL risk, as shown in Supplementary Figure 1. We found no significant association between *SOX1* promoter methylation with LSIL risk. Then, eight studies including 427 patients with HSIL and 896 controls were included to assess the effect of *SOX1* promoter methylation on HSIL risk. As shown in Figure 2, *SOX1* promoter hypermethylation was associated with a 4.20-fold (95% CI: 2.98–5.93, *p* < 0.001) increased HSIL risk. This association remained significant in all subgroups, and no significant heterogeneity was found in all comparisons.

**Figure 3:**
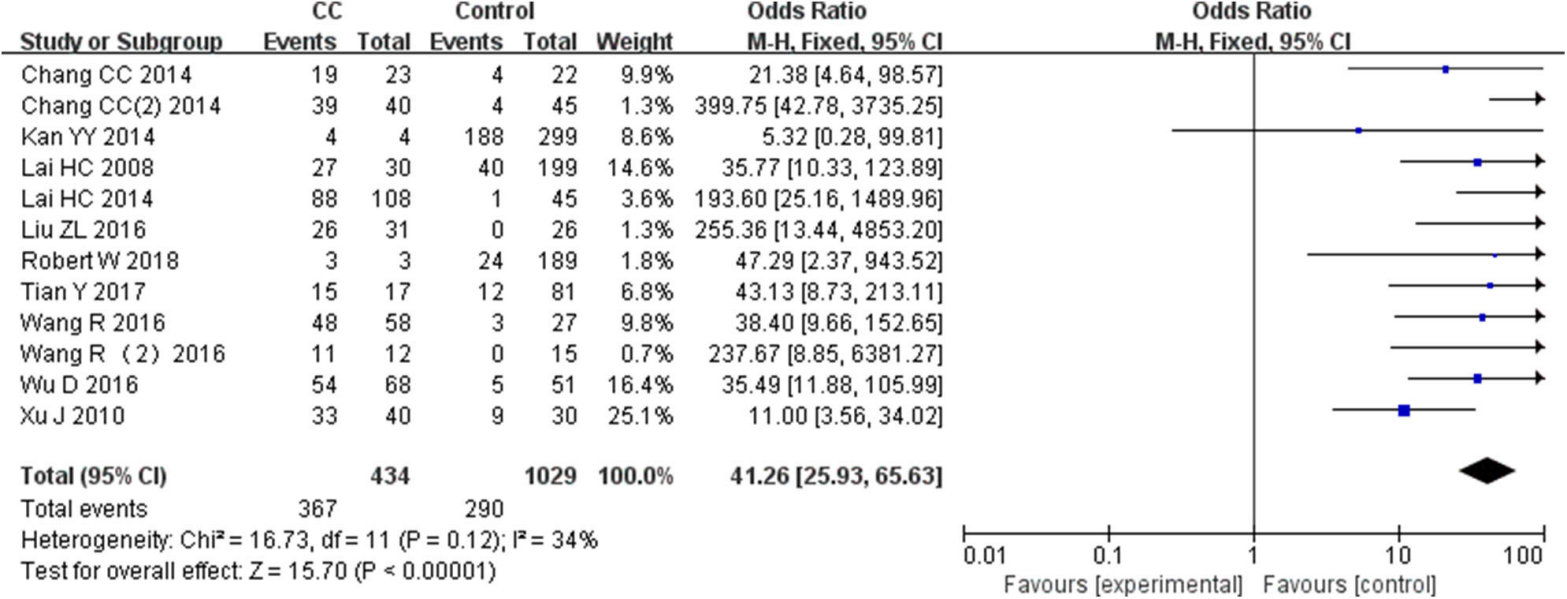
Forest plots for associations of *SOX1* promoter hypermethylation with the risk of cervical cancer. The squares represent the ORs for individual studies. The size of the square reflects the weight of included studies. Bars represent the 95% confidence intervals (CIs). The center of the diamond represents the summary effect size.

Twelve studies including 434 patients with CC and 1,029 controls were included to assess the effect of *SOX1* promoter methylation on CC risk. As shown in Figure 3, *SOX1* promoter hypermethylation was associated with a 41.26-fold (95% CI: 25.93–65.63, *p* < 0.001) increased CC risk. In consideration that mild heterogeneity was observed in the overall comparison (*I*^2^= 34%), subgroup analyses (Table 3), meta-regression, and Galbraith plot were performed to seek the potential sources of heterogeneity. *I*^2^ decreased to 0 in the subgroups of “Caucasian,” “non-healthy,” “exfoliated cells,” and “publication year after 2015.” *SOX1* promoter hypermethylation was significantly associated with CC risk in all subgroups. Results of meta-regression showed that none of the subgroups above were major sources of heterogeneity (Supplementary Table 3). Moreover, a Galbraith plot was further depicted, spotting two outliers as major sources of heterogeneity (Supplementary Figure 3). These two studies (Lai et al., 2010; Kan et al., 2014) were both classified into “exfoliated cells,” “publication year before 2015,” and low-quality studies, and exclusion of these two studies led to a decrease in *I*^2^-value from 34 to 0%.

**Table 3:** Pooled results for the association of *SOX1* promoter hypermethylation with cervical cancer risk.

**Comparisons**	**Studies (*N*)**	**Sample size (CC/Control)**	**Heterogeneity**		**Model^a^**	**Effect size**	
			***I*^**2**^(%)**	***P*_**Q-text**_**		**OR (95% CI)**	***P***
Total	12	434/1029	34	0.12	F	41.26 (25.93–65.63)	<0.001
Ethnicity							
Asian	9	361/798	48	0.05	F	39.84 (24.05–66.00)	<0.001
Caucasian	3	73/231	0	0.60	F	51.33 (16.22–162.41)	<0.001
Source of controls							
Healthy	5	206/437	57	0.05	R	74.64 (16.11–345.78)	<0.001
Non-healthy^b^	7	228/592	0	0.53	F	30.66 (17.97–52.32)	<0.001
Materials							
Tissue	3	160/141	70	0.02	R	52.71 (11.63–238.84)	<0.001
Exfoliated cells	9	274/888	0	0.49	F	45.54 (24.12–85.97)	<0.001
Publication year							
≥ 2015	6	189/389	0	0.76	F	49.95 (25.28–98.70)	<0.001
< 2015	6	245/640	63	0.02	R	36.89 (11.91–114.30)	<0.001
Quality of studies							
High (>11)	7	183/578	0	0.45	F	48.59 (25.95–90.99)	<0.001
Low (≤11)	5	251/451	60	0.04	R	35.22 (10.04–123.47)	<0.001

a *When significant heterogeneity was found (I^2^≥50% or P_Q−test_≤0.1), a random-effects model with the inverse variance method was used to pool the results; otherwise, a fixed-effects model was applied*.

b *Non-healthy controls included autologous controls and controls with benign gynecological diseases*.

*N, number; F, fixed-effects model; R, random-effects model*.

**Figure 4:**
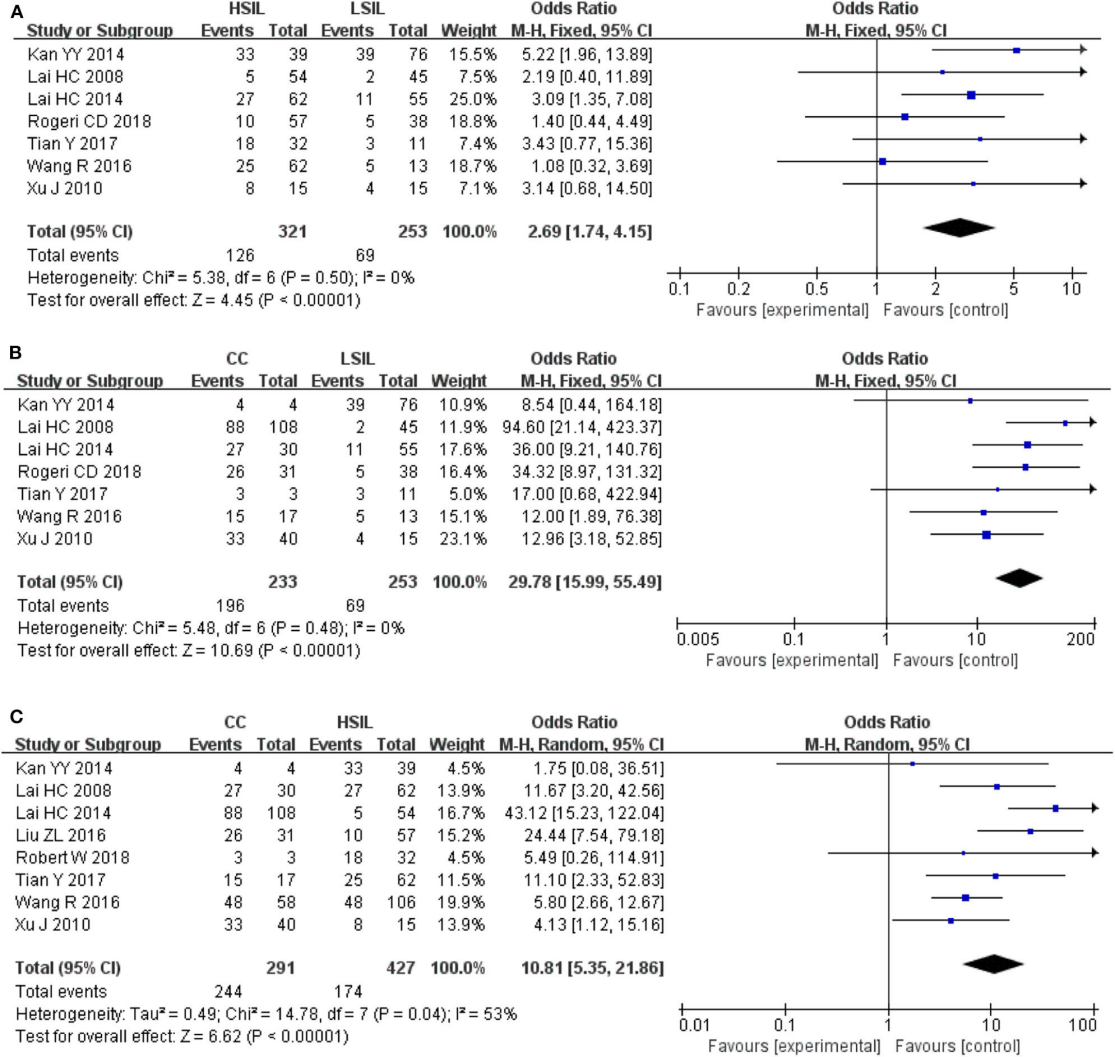
**(A)** Forest plots for associations of *SOX1* promoter hypermethylation with the risk of HSIL compared with LSIL; **(B)** Forest plots for associations of *SOX1* promoter hypermethylation with the risk of LSIL compared with cervical cancer; **(C)** Forest plots for associations of *SOX1* promoter hypermethylation with the risk of HSIL compared with cervical cancer. The squares represent the ORs for individual studies. The size of the square reflects the weight of included studies. Bars represent the 95% confidence intervals (CIs). The center of the diamond represents the summary effect size.

To explore whether the level of methylation could be served as a biomarker to differentiate LSIL, HSIL or CC, comparison between each other were performed. Seven studies including 253 patients with LSIL and 321 HSIL were included to assess the effect of *SOX1* promoter hypermethylation in HSIL compared with LSIL. As shown in Figure 4A and Table 4, *SOX1* promoter hypermethylation was associated with a 2.69-fold (95% CI: 1.74-4.15, *p* < 0.001) increased risk in HSIL compared with LSIL with a low level of heterogeneity (*I*^2^= 0%). Seven studies including 253 patients with LSIL and 233 CC were included to assess the effect of *SOX1* promoter hypermethylation in CC compared with LSIL. As shown in Figure 4B and Table 4, *SOX1* promoter hypermethylation was associated with a 29.78-fold (95% CI: 15.99-55.49, *p* < 0.001) increased risk in CC compared with LSIL with a low level of heterogeneity (*I*^2^= 0%). As shown in Figure 4C and Table 4, *SOX1* promoter hypermethylation was associated with a 10.81-fold (95% CI: 5.35–21.86, *p* < 0.001) increased risk in CC compared with HSIL with a moderate level of heterogeneity (*I*^2^= 53%). In order to seek the potential sources of heterogeneity, a Galbraith plot was further depicted, spotting one outlier as major sources of heterogeneity (Supplementary Figure 4). The exclusion of the study (Lai et al., 2014) led to a decrease in *I*^2^-value from 34 to 0%.

**Table 4:** Pooled results for the association of *SOX1* promoter hypermethylation and comparison between LSIL, HSIL and CC.

**Comparisons**	**Studies (*N*)**	**Patients (*N*)**	**Heterogeneity**		**Model^a^**	**Effect size**	***P***
			***I*^**2**^(%)**	***P*_**Q-text**_**		**OR (95% CI)**	
LSIL vs. HSIL	7	574	0	0.50	F	2.69 (1.74–4.15)	<0.001
LSIL vs. CC	7	486	0	0.48	F	29.78 (15.9–55.49)	<0.001
HSIL vs. CC	8	718	53	0.04	R	10.81 (5.35–21.86)	<0.001

a *When significant heterogeneity was found (I^2^≥50% or P_Q−test_ ≤ 0.1), a random-effects model with the inverse variance method was used to pool the results; otherwise, a fixed-effects model was applied*.

**Figure 5:**
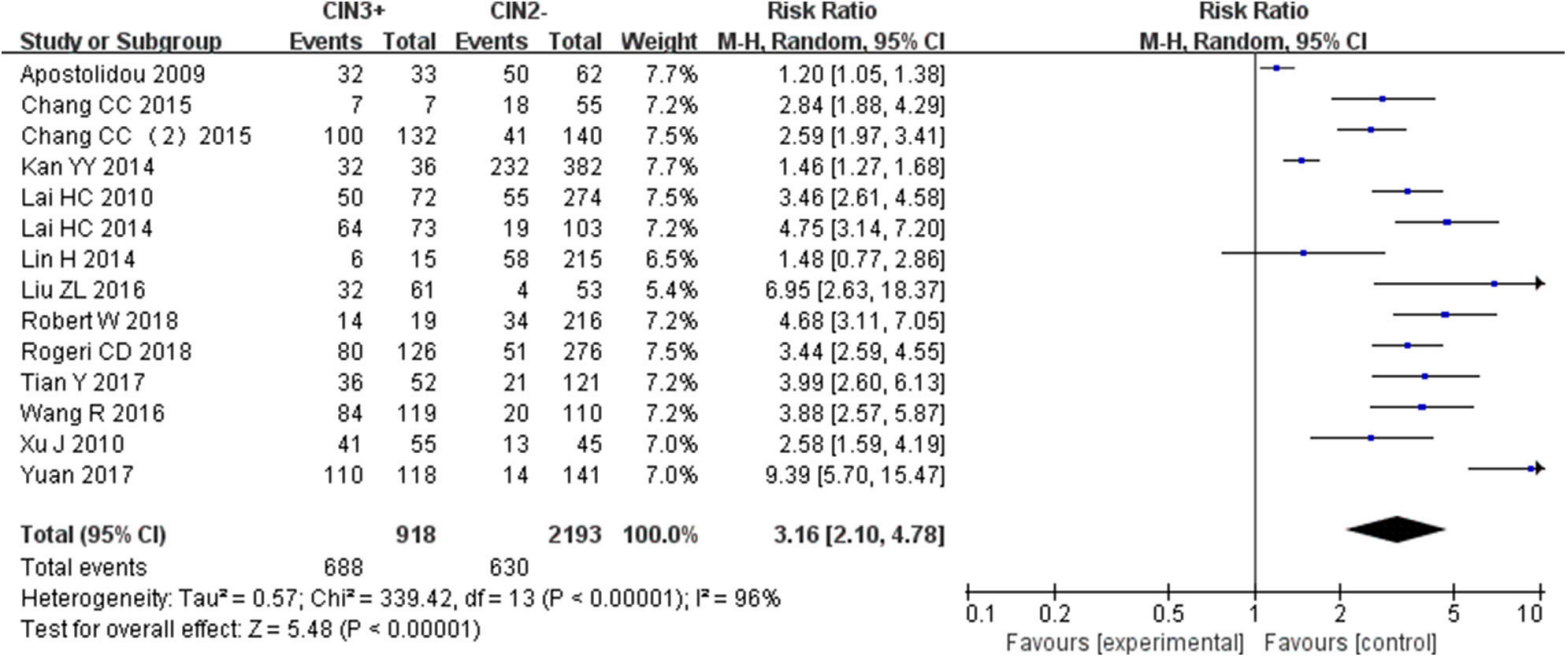
Forest plots for associations of *SOX1* promoter hypermethylation with the risk of CIN3+. The squares represent the ORs for individual studies. The size of the square reflects the weight of included studies. Bars represent the 95% confidence intervals (CIs). The center of the diamond represents the summary effect size.

Fourteen studies including 918 patients with CIN3+ and 2193 CIN2– were included to assess the effect of *SOX1* promoter methylation on CIN3+ risk. As shown in Figure 5, *SOX1* promoter hypermethylation was associated with an 3.16-fold (95% CI: 2.10-4.78, *p* < 0.001) increased CIN3+ risk. In consideration that high heterogeneity was observed in the overall comparison (*I*^2^= 75%), subgroup analyses (Table 5) and Galbraith plot were performed to seek the potential sources of heterogeneity. *I*^2^ was decreased to 0 in the subgroups of “Other ethnicity” and “materials of tissue.” In addition, *SOX1* promoter hypermethylation was significantly associated with CIN3+ risk in all subgroups.

**Table 5:** Pooled results for the association of *SOX1* promoter hypermethylation with CIN3+ risk.

**Comparisons**	**Studies (*N*)**	**Sample size (CIN3+/CIN2–)**	**Heterogeneity**		**Model^a^**	**Effect size**	
			***I*^**2**^(%)**	***P*_**Q-text**_**		**OR (95% CI)**	***P***
Total	14	918/2193	75	<0.001	R	11.12 (7.04–17.55)	<0.001
Ethnicity							
Asian	10	621/1529	82	<0.001	R	11.67 (6.05–22.51)	<0.001
Others	4	297/664	0	0.93	F	9.14 (6.42–13.01)	<0.001
Source of controls							
Healthy	4	302/678	69	0.02	R	11.30 (5.21–24.52)	<0.001
Non-healthy^b^	10	616/1515	79	<0.001	R	11.07 (6.09–20.12)	<0.001
Materials							
Tissue	2	62/100	0	0.34	F	8.92 (3.87–20.53)	<0.001
Exfoliated cells	12	856/2093	79	<0.001	R	11.28 (6.86–18.55)	<0.001
Publication year							
≥ 2015	8	634/1112	79	0.56	R	14.60 (7.90–26.96)	<0.001
< 2015	6	284/1081	72	0.003	R	7.56 (3.58–15.96)	<0.001
Quality of studies							
High (>11)	8	600/1296	32	0.17	F	3.49 (3.07–3.97)	<0.001
Low (≤11)	6	318/898	98	<0.001	R	2.65 (1.24–5.68)	0.01

a *When significant heterogeneity was found (I^2^≥50% or P_Q−test_ ≤ 0.1), a random-effects model with the inverse variance method was used to pool the results; otherwise, a fixed-effects model was applied*.

b *Non-healthy controls included autologous controls and controls with benign gynecological diseases*.

*N, number; F, fixed-effects model; R, random-effects model*.

### Diagnostic Performance of CC and CIN3+

**Figure 6:**
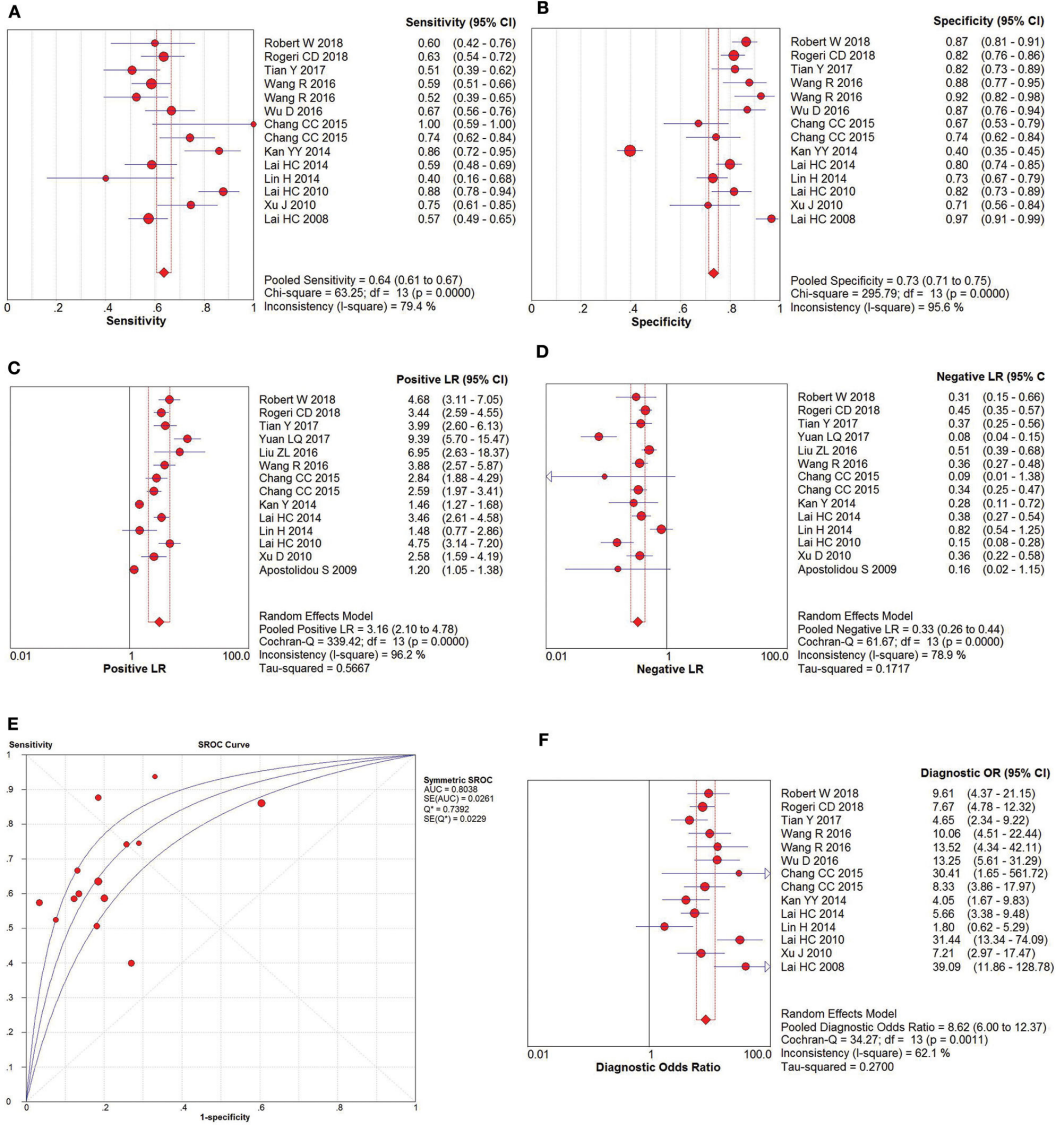
SROC curve **(E)** and forest plots of sensitivity **(A)**, specificity **(B)**, positive-likelihood ratio (PLR) **(C)**, negative-likelihood ratio (NLR) **(D)** and diagnostic odds ratio (DOR) **(F)** of *SOX1* hypermethylation in the diagnosis of cervical cancer.

The pooled accuracies for *SOX1* methylation were determined to evaluate their usefulness as a biomarker for screening patients with CC. As shown in Figure 6, the area under the curve (AUC) was 0.925, pooled sensitivity was 0.85 (95% CI: 0.81–0.88), and pooled specificity was 0.72 (95% CI: 0.69–0.75). Meanwhile, the PLR of *SOX1* hypermethylation was 6.29 (95% CI: 3.34–11.85), and the NLR was 0.19 (95% CI: 0.15–0.24). Our analysis showed that *SOX1* hypermethylation could be a useful biomarker for CC (DOR = 41.05, 95% CI: 22.07–76.36).

**Figure 7:**
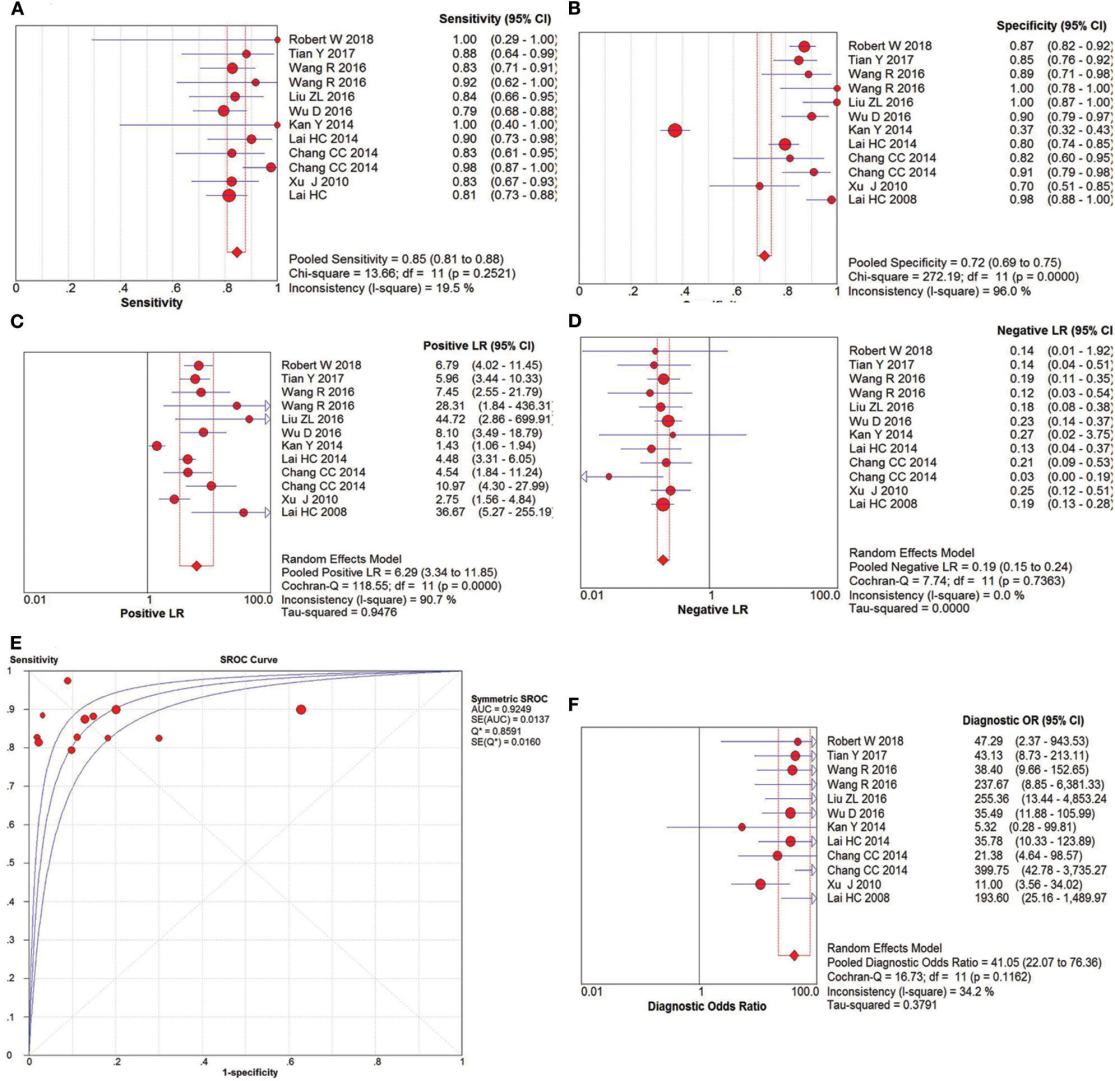
SROC curve **(E)** and forest plots of sensitivity **(A)**, specificity **(B)**, positive-likelihood ratio (PLR) **(C)**, negative-likelihood ratio (NLR) **(D)** and diagnostic odds ratio (DOR) **(F)** of *SOX1* hypermethylation in the diagnosis of CIN3+.

The pooled accuracies for *SOX1* methylation were determined to evaluate their usefulness as a biomarker for screening patients with CIN3+. As shown in Figure 7, the AUC was 0.8038, pooled sensitivity was 0.64 (95% CI: 0.61–0.67), and pooled specificity was 0.73 (95% CI: 0.71–0.75). Meanwhile, the PLR of *SOX1* hypermethylation was 3.16 (95% CI: 2.10–4.78), and the NLR was 0.33 (95% CI: 0.26–0.44). Our analysis showed that *SOX1* hypermethylation could be a useful biomarker for CIN3+ (DOR = 8.62, 95% CI: 6.00–12.37).

### Validation by Online Databases

**Figure 8:**
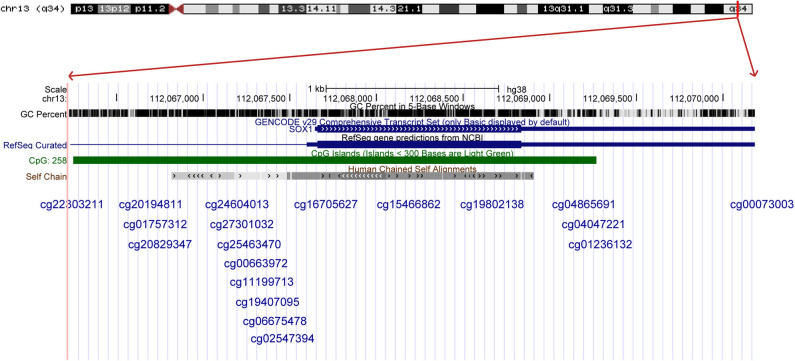
The location of all the CpG island probes in the *SOX1*.

**Figure 9:**
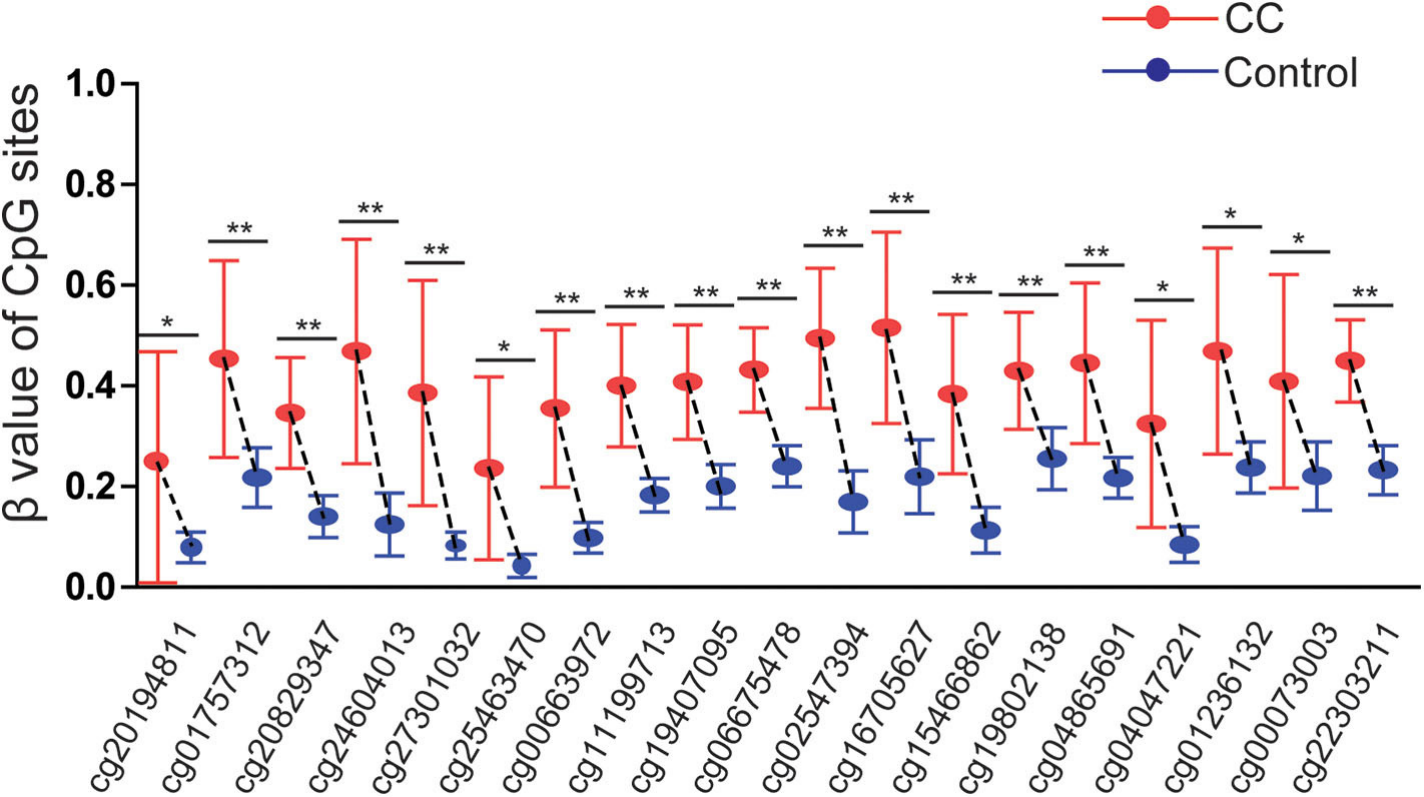
Significant differences in 19 CpG sites of *SOX1* between cervical cancer and controls in TCGA and GEO dataset. *P*-value were calculated by the Mann–Whitney *U*-test. **P < 0.001,*P < 0.05.

The results were validated by quantitative methylation data from online databases, including TCGA and GEO. Six datasets, including TCGA CESE, GSE99511, GSE46306, GSE41384, GSE36637, and GSE30760, involved genome-wide quantitative methylation data of 73 controls, 69 patients with CC, and 218 patients with SIL. A total of 19 CpG sites (cg20194811, cg01757312, cg20829347, cg24604013, cg27301032, cg25463470, cg00663972, cg11199713, cg19407095, cg06675478, cg02547394, cg16705627, cg15466862, cg19802138, cg04865691, cg04047221, cg01236132, cg00073003, and cg22303211) in the promoter region of *SOX1* were included, and the location of these CpG island probes was depicted as shown in Figure 8. All CpG sites showed significant results with *p* < 0.05 when the methylation level of CC was compared with that of the controls, supporting the effect of *SOX1* promoter hypermethylation in CC (Figure 9). All CpG sites showed great diagnostic value for CC with AUC from 0.799 to 0.983, as shown in Table 6. Furthermore, we identified top 10 CpG sites (cg20829347, cg24604013, cg00663972, cg11199713, cg19407095, cg06675478, cg02547394, cg15466862, cg04047221, and cg22303211) with excellent diagnostic values for CC with AUCs from 0.928 to 0.983, sensitivities from 0.923 to 0.946, and specificities from 0.922 to 1, which are shown in bold in Table 6.

**Table 6:** Diagnostic value of 19 CpG sites of *SOX1* promoter for cervical cancer.

**CpG Sites**	**Location^a^**	**Position to TSS^a^**	**CpG Feature Type ^a^^,^^b^**	**Studies N^c^**	**Sample size (CC/controls)**	**Diagnostic value of CpG sites for CC**				***P*-value**
						**Cut-off β value**	**Sensitivity (%)**	**Specificity (%)**	**AUC**	
cg20194811	chr13:112066177	−1471	N_Shore	3	13/51	0.130	0.615	0.980	0.799	2.817E-02
cg01757312	chr13:112066251	−1397	Island	3	13/51	0.360	0.760	1.000	0.890	9.348E-04
**cg20829347**	**chr13:112066613**	–**1035**	**Island**	**3**	**13/51**	**0.215**	**0.923**	**0.961**	**0.928**	**1.770E-05**
**cg24604013**	**chr13:112067011**	–**637**	**Island**	**3**	**13/51**	**0.203**	**0.923**	**0.980**	**0.934**	**1.165E-04**
cg27301032	chr13:112067111	−537	Island	3	13/51	0.146	0.846	1.000	0.923	3.770E-04
cg25463470	chr13:112067398	−250	Island	3	13/51	0.072	0.923	0.980	0.934	2.362E-03
**cg00663972**	**chr13:112067411**	–**237**	**Island**	**3**	**13/51**	**0.161**	**0.923**	**0.980**	**0.958**	**6.590E-05**
**cg11199713**	**chr13:112067459**	–**189**	**Island**	**3**	**13/51**	**0.300**	**0.923**	**1.000**	**0.940**	**3.030E-05**
**cg19407095**	**chr13:112067464**	–**184**	**Island**	**3**	**13/51**	**0.297**	**0.923**	**0.980**	**0.941**	**2.260E-05**
**cg06675478**	**chr13:112067472**	–**176**	**Island**	**6**	**69/73**	**0.362**	**0.939**	**0.973**	**0.959**	**1.740E-06**
**cg02547394**	**chr13:112067475**	–**173**	**Island**	**3**	**13/51**	**0.346**	**0.923**	**1.000**	**0.965**	**1.470E-06**
cg16705627	chr13:112067636	−12	Island	3	13/51	0.364	0.769	0.980	0.920	1.020E-04
**cg15466862**	**chr13:112068019**	**371**	**Island**	**3**	**13/51**	**0.171**	**0.923**	**0.922**	**0.976**	**4.430E-05**
cg19802138	chr13:112068405	757	Island	3	13/51	0.314	0.923	0.902	0.902	1.367E-04
cg04865691	chr13:112069107	1459	Island	3	13/51	0.320	0.846	1.000	0.938	2.346E-04
**cg04047221**	**chr13:112069163**	**1515**	**Island**	**3**	**13/51**	**0.142**	**0.923**	**0.961**	**0.932**	**1.212E-03**
cg01236132	chr13:112069267	1619	Island	3	13/51	0.283	0.846	0.863	0.882	1.539E-03
cg00073003	chr13:112070269	2621	S_Shore	3	13/51	0.302	0.769	0.922	0.833	7.915E-03
**cg22303211**	**chr13:111771727**	**–**	**Island**	**3**	**56/22**	**0.338**	**0.946**	**1.000**	**0.983**	**4.690E-05**

a *Information for position of CpG site according to TCGA data*.

b *Island is the start coordinates of CpG island; N_Shore is 0-2 kb upstream from the position of CpG island; S_Shore is 0-2 kb downstream from the position of CpG island*.

c *TCGA, GSE99511 and GSE46306 used the Illumina HumanMethylation 450K BeadChip, which included methylation probes of all CpG island except cg22303211 in SOX1 promoter; GSE41384, GSE36637, and GSE30760 used the Illumina HumanMethylation 27K BeadChip, which included methylation probes of cg06675478 and cg22303211*.

**Figure 10:**
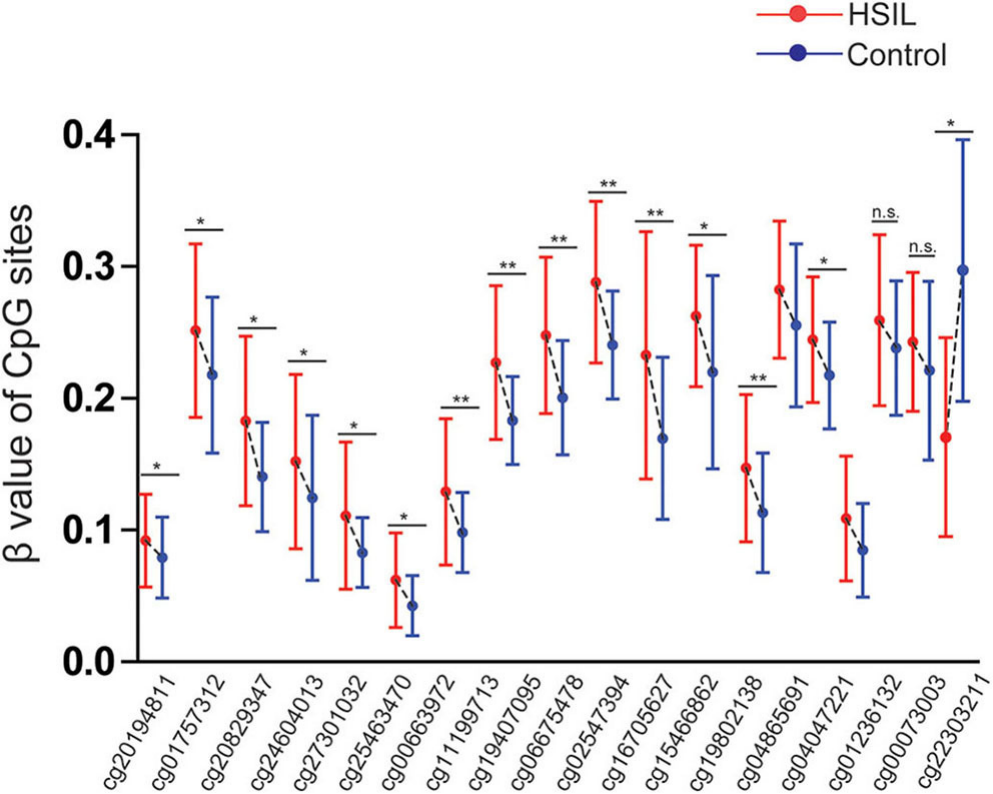
Significant differences in 19 CpG sites of *SOX1* between HSIL and controls in TCGA and GEO dataset. *P*-value were calculated by the Mann–Whitney *U*-test. *P < 0.05, **P < 0.001. If *P* > 0.05, there is no significance.

Three datasets including GSE99511, GSE46306, and GSE41384 involved quantitative methylation data of 51 controls and 53 patients with HSIL. Of 19 CpG sites, 16 showed significant results with *p* < 0.05 and 6 showed significant results with *p* < 0.001 when the methylation level of HSIL was compared with that of the controls, supporting the effect of *SOX1* promoter hypermethylation in HSIL (Figure 10). Moreover, we identified two CpG sites (cg11199713 and cg19407095) with great diagnostic values for HSIL with AUCs from 0.725 to 0.729, sensitivities from 0.804 to 0.824, and specificities of 0.623, which are shown in bold in Table 7.

**Table 7:** Diagnostic value of 19 CpG sites of *SOX1* promoter for HSIL.

**CpG Sites**	**Location^a^**	**Position to TSS^a^**	**CpG Feature Type^a^^,^^b^**	**Studies N^c^**	**Sample size (CC/controls)**	**Diagnostic value of CpG sites for HSIL**				***P*-value**
						**Cut-off β value**	**Sensitivity (%)**	**Specificity (%)**	**AUC**	
cg20194811	chr13:112066177	−1471	N_Shore	2	53/48	0.056	0.392	0.830	0.605	0.048
cg01757312	chr13:112066251	−1397	Island	2	53/48	0.222	0.608	0.679	0.648	0.007
cg20829347	chr13:112066613	−1035	Island	2	53/48	0.191	0.922	0.453	0.683	1.26E-04
cg24604013	chr13:112067011	−637	Island	2	53/48	0.171	0.863	0.340	0.616	0.032
cg27301032	chr13:112067111	−537	Island	2	53/48	0.091	0.608	0.660	0.676	0.002
cg25463470	chr13:112067398	−250	Island	2	53/48	0.062	0.824	0.528	0.674	0.001
cg00663972	chr13:112067411	−237	Island	2	53/48	0.136	0.941	0.358	0.673	0.001
**cg11199713**	**chr13:112067459**	–**189**	**Island**	**2**	**53/48**	**0.208**	**0.804**	**0.623**	**0.729**	**8.27E-06**
**cg19407095**	**chr13:112067464**	–**184**	**Island**	**2**	**53/48**	**0.228**	**0.824**	**0.623**	**0.725**	**1.09E-05**
cg06675478	chr13:112067472	−176	Island	3	63/51	0.302	0.961	0.453	0.721	1.03E-05
cg02547394	chr13:112067475	−173	Island	2	53/48	0.153	0.490	0.830	0.701	1.03E-04
cg16705627	chr13:112067636	−12	Island	2	53/48	0.249	0.647	0.642	0.670	1.06E-03
cg15466862	chr13:112068019	371	Island	2	53/48	0.108	0.569	0.774	0.686	9.41E-04
cg19802138	chr13:112068405	757	Island	2	53/48	0.276	0.745	0.585	0.642	0.017
cg04865691	chr13:112069107	1459	Island	2	53/48	0.252	0.843	0.396	0.646	0.002
cg04047221	chr13:112069163	1515	Island	2	53/48	0.091	0.529	0.717	0.632	0.004
cg01236132	chr13:112069267	1619	Island	2	53/48	0.239	0.588	0.660	0.609	0.067
cg00073003	chr13:112070269	2621	S_Shore	2	53/48	0.217	0.490	0.736	0.584	0.072
cg22303211	chr13:111771727	–	Island	1	10/3	0.282	0.700	1.000	0.733	0.063

a *Information for position of CpG site according to TCGA data*.

b *Island is the start coordinates of CpG island; N_Shore is 0-2 kb upstream from the position of CpG island; S_Shore is 0-2 kb downstream from the position of CpG island*.

c *GSE99511 and GSE46306 used the Illumina HumanMethylation 450K BeadChip, which included methylation probes of all CpG island except cg22303211 in SOX1 promoter; GSE41384 used the Illumina HumanMethylation 27K BeadChip, which included methylation probes of cg06675478 and cg22303211*.

### Trial Sequential Analysis

**Figure 11:**
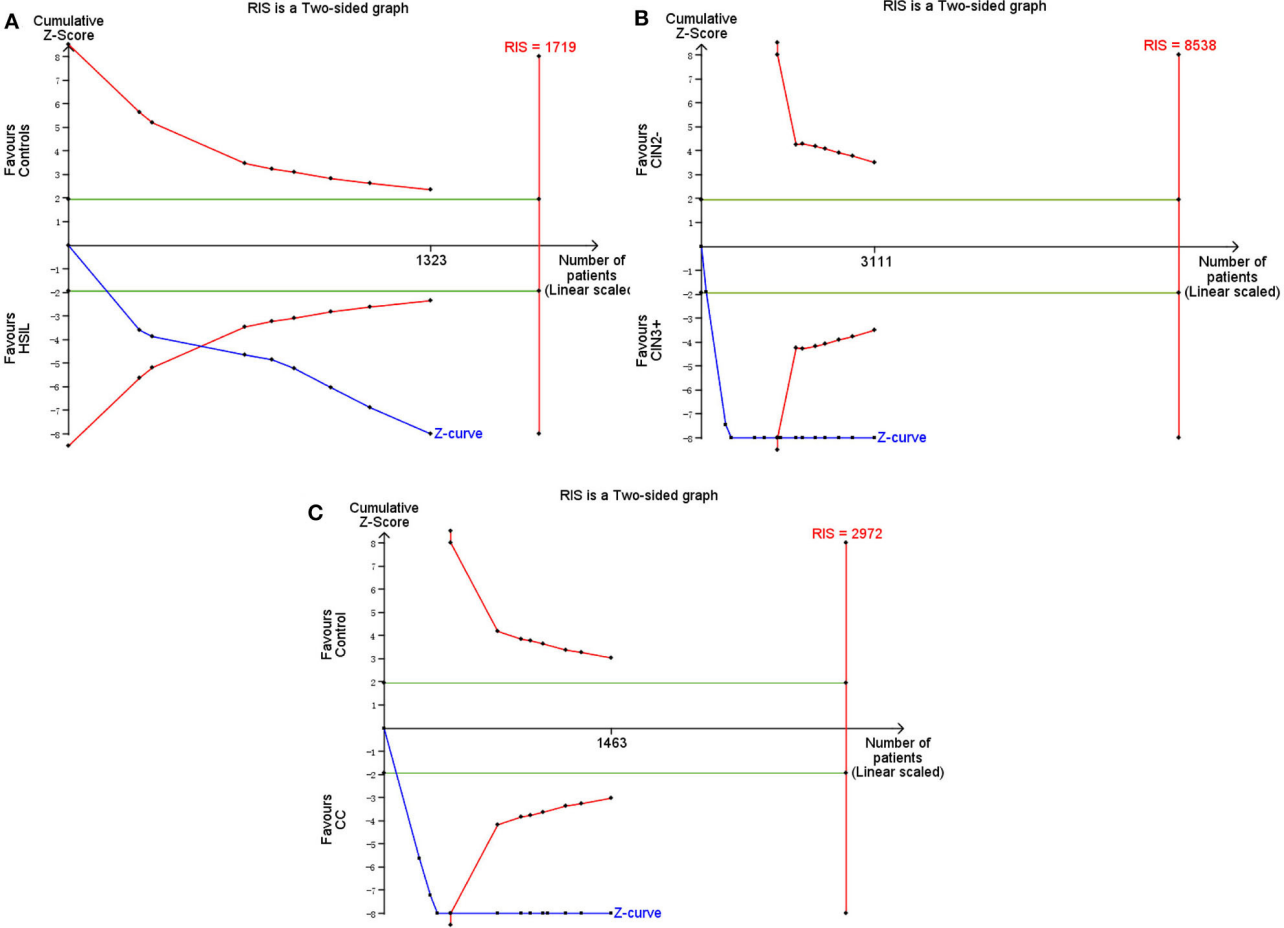
Trial sequential analysis estimating the required sample information in relation to HSIL **(A)**/CC **(C)** compared to controls, and CIN3+ compared to CIN2–**(B)**.

TSA was performed to evaluate the stability of results and estimate RIS. When HSIL (the estimated required sample size of 1,719 cases: Figure 11A) and CC (the estimated required sample size of 2,972 cases: Figure 11C) were compared with controls, and CIN3+ were compared with CIN2– (the estimated required sample size of 8,538 cases: Figure 11B), the cumulative Z-curve crossed the conventional boundary and the trial sequential monitoring boundary but not RIS, which indicated that the size was sufficient and significant associations were observed.

### Sensitivity Analysis and Publication Bias

In sensitivity analysis (Supplementary Figure 6), sequential removal of each study had no significant impact on the pooled results. In all comparisons, the shapes of funnel plots (Supplementary Figure 7) were symmetric and the values of the Egger's test were larger than 0.05, indicating no significant publication bias in this meta-analysis.

## Discussion

SOX genes are a family possessing an HMGbox (SRY-related-high mobility group box) DNA binding domain. They are involved in the coding of transcription factors during embryonic development and apoptosis (Ekonomou et al., 2005). SOX genes are related to the Wnt/β-catenin signaling pathway. The aberrant expression of SOX genes could activate the β-catenin signaling pathway, promote β-catenin breakdown, and inhibit the activity of β-catenin, thus affecting embryonic development and tumor formation (Kormish et al., 2010). The *SOX1* gene, a member of the SOX gene family, is involved in the development and progression of various tumors. For instance, *SOX1* methylation is associated with liver cancer, and *SOX1* promoter methylation level is significantly higher in patients with high TNM stage (stages III and IV) than in those with low TNM stage. The *SOX1* methylation level is significantly higher in liver cancer patients with large tumor size (≥5 cm) than in those with small tumor size (<3 cm) (Liu et al., 2017). In addition, *SOX1* methylation is a useful biomarker for oral squamous cell carcinoma (Cheng et al., 2016) and CC screening (Huang et al., 2017). Another study reported that *SOX1* gene methylation is associated with the prognosis prediction of bladder cancer (Lopez et al., 2017). At present, many studies explored the correlation between *SOX1* gene promoter and cervical cancer and SILs, but the research results are inconsistent.

Seventeen articles were included in this meta-analysis, however two articles (Chang et al., 2014; Wang et al., 2016) each article contains two studies involved tissues and cervical exfoliated cells, respectively. In total, 19 studies involving 3,491 patients with CC or pre-carcinoma and 1,029 controls were included in this meta-analysis. We found an increasing trend of *SOX1* hypermethylation rates with the development of carcinogenesis from LSIL to HSIL and eventually to CC. Compared with the control specimens, *SOX1* promoter hypermethylation progressively increased the risk of HSIL by 4.20-fold (*p* < 0.001) and CC by 41.26-fold (*p* < 0.001). This epigenetic effect of *SOX1* above was further verified by extracting quantitative methylation data from the TCGA and GEO databases. We found that all CpG sites had higher methylation levels in CC than in the controls. And most CpG sites had higher methylation levels in HSIL than in the controls. These results illustrate that *SOX1* promoter hypermethylation is correlated with the progression of SILs to cervical carcinoma, which is consistent with previous studies (Kan et al., 2014; Lai et al., 2014; Wang et al., 2016). In addition, Chen et al. (2016) reported that hypermethylation of *SOX1* combined with *PAX1* showed highly sensitive biomarker for the detection of CC, the sensitivity and specificity were 0.72 and 0.86, respectively. Moreover, we identified top 10 CpG sites with high diagnostic value in CC. Thus, primers based on these 10 loci could be designed to improve the diagnostic accuracy for CC in future research.

Moreover, 14 relevant studies including 918 patients with CIN3+ and 2193 CIN2– showed an AUC of 0.838 in the SROC curve, illustrating that *SOX1* promoter hypermethylation could distinguish CIN3+ patients from CIN2– to a certain extent. The pooled sensitivity and specificity were 0.75 and 0.71, respectively. DOR is a great indicator to assess the accuracy that synthesized the data from sensitivity and specificity (Glas et al., 2003). In specific, DOR larger than 1 shows good discrimination of the index test, and the higher the better (Bohning et al., 2011). A DOR of 11.12 in this meta-analysis showed a great diagnostic value of *SOX1* methylation for screening CIN3+. Thus, we conclude that the promoter hypermethylation of *SOX1* or combined with another TSG could be a great biomarker for screening CIN3+. These results are consistent with previous studies. In Lai's research (Lai et al., 2014), methylation of *SOX1* was a potential new biomarker for the detection of CIN3+ with a methylation rate of 100% in cases with CIN3+. Moreover, Tian et al. (2017) reported that hypermethylation of *SOX1* combined with either *PAX1* or *ZNF582* is a highly sensitive biomarker for the detection of CIN3+. Similarly, Robert et al. (2019) reported that promoter methylation of *SOX1* combined with *ZSCAN1* shows a higher specificity for screening of CIN3+ than the two other marker panels.

Furthermore, this meta-analysis explored whether the level of methylation could be served as a biomarker to differentiate LSIL, HSIL, or CC for the first time. *SOX1* promoter hypermethylation was associated with a 2.69 and 29.78–fold increased risk in HSIL and CC compared with that of LSIL, respectively. And, *SOX1* promoter hypermethylation was associated with a 10.81-fold increased risk in CC compared with HSIL. It suggested that the level of methylation could also be served as a biomarker to differentiate LSIL, HSIL or CC, that would carry stronger clinical implications in the early detection of cervical cancer.

No significant heterogeneity was found in total or in all subgroups for the association of *SOX1* methylation with HSIL. However, mild and high heterogeneity were observed in our meta-analysis for the association of *SOX1* methylation with CC. Thus, the results of the association of *SOX1* methylation with CC were first pooled by the fixed-effect model, whereas the association of *SOX1* methylation with CC was first pooled by the random-effect model, which conservatively estimates the article weights after adjusting for inter-study variances (DerSimonian and Kacker, 2007). Then, the potential sources of heterogeneity were explored by three statistical approaches, including subgroup analysis and meta-regression, to identify the complex factors associated with observed heterogeneity, and then Galbraith plots were depicted to explore the contributions of individual studies to overall heterogeneity. In the comparison between *SOX1* promoter hypermethylation and CC risk, the results of subgroup analysis showed that studies of Asians, healthy individuals as controls, materials from tissue, and publication year before 2015 were probably the sources of heterogeneity. Galbraith plots spotted two outliers (Kan et al., 2014; Lai et al., 2014) as major sources of heterogeneity, and these two studies were both classified into “exfoliated cells,” “publication year before 2015,” and low-quality studies. Moreover, the hypermethylation rate of the control group in one of these two studies was 62.88%, which is much higher than that of the control group in overall (20.80%), whereas that of the other studies (2.22%) was much lower than that of the control group in overall, indicating the existence of inter-study differences.

There are some innovations in this meta-analysis. This study is the first to evaluate the trend of methylation frequencies of *SOX1* in different carcinogenesis stages from precancerous lesions to CC in a meta-analysis. In addition, it involved Caucasians and Asians for the first time. Moreover, the diagnostic value of *SOX1* hypermethylation to be a biomarker for screening CC or CIN3+ was evaluated in this meta-analysis for the first time. Finally, TSA was performed to evaluate the stability of results and estimate RIS, the results of which showed that the size was sufficient and significant associations were observed.

However, this meta-analysis still has some limitations. First, the role of *SOX1* promoter hypermethylation in the clinicopathological features of CC lacks appraisal. Only investigated in the FIGO stage, results showed no significant difference between *SOX1* promoter methylation in the low (stage I/II) and high (stage III/IV) stages of CC (Supplementary Figure 2). This result can be ascribed to the insufficient data about the clinicopathological features of CC. In included studies of this meta-analysis, only two studies involved the histological type of CC, HPV infection, or age, and only one study involved size of tumor or lymph node metastasis. Additional studies about the role of *SOX1* promoter hypermethylation in the clinicopathological features of CC are needed in the future. Second, the pooled results were only performed in Asians, Caucasians, and Brazilians, in this meta-analysis. Further studies involving Africans are still needed. Third, only full-text articles written in English or Chinese were included. Articles in other languages were excluded because of unreadable contents or insufficient data, thereby causing a selection bias.

In conclusion, the pooled results in this meta-analysis illustrated that DNA methylation of *SOX1* could be a promising biomarker for screening CC, high-grade squamous intraepithelial lesion, and CIN3+.

## Data Availability Statement

All datasets generated for this study are included in the article/Supplementary Material.

## Author Contributions

H-ZT contributed to the design and final approval of the study. HG contributed to performing the data analyses. JH contributed to interpretation of data, completion of figures and tables, and writing the paper. All authors have read and approved the final manuscript.

## Conflict of Interest

The authors declare that the research was conducted in the absence of any commercial or financial relationships that could be construed as a potential conflict of interest.

## Abbreviations

AdC: adenocarcinoma
AUC: area under the curve
CC: cervical cancer
CIN2-: cervical intraepithelial neoplasia II or less
CIN3+: cervical intraepithelial neoplasia grade III or worse
CIS: carcinoma *in situ*
CIs: confidence intervals
DOR: diagnostic odds ratio
FIGO: federation International of Gynecology and Obstetr
HSIL: high-grade squamous intra-epithelial lesion
Island: the start coordinates of CpG island
LSIL: low-grade squamous intra-epithelial lesion
NLR: negative likelihood ratio
N_Shore: 0-2 kb upstream from the position of CpG island
OR: odd ratio
PLR: positive likelihood ratio
PRISMA: Preferred Reporting Items for Systematic Reviews and Meta-Analysis
QMSP: quantitative methylation-specific PCR
RIS: required information size
SCC: squamous cell carcinoma
SROC: summary receiver operator characteristic
S_Shore: 0-2 kb downstream from the position of CpG island
TSGs: tumor suppressor genes
TSS: transcription start sites.
